# PCSK9 activation promotes early atherosclerosis in a vascular microphysiological system

**DOI:** 10.1063/5.0167440

**Published:** 2023-10-16

**Authors:** Jounghyun H. Lee, Kevin L. Shores, Jason J. Breithaupt, Caleb S. Lee, Daniella M. Fodera, Jennifer B. Kwon, Adarsh R. Ettyreddy, Kristin M. Myers, Benny J. Evison, Alexandra K. Suchowerska, Charles A. Gersbach, Kam W. Leong, George A. Truskey

**Affiliations:** 1 Department of Biomedical Engineering, Columbia University, New York, New York 10032, USA; 2 Department of Biomedical Engineering, Duke University, Durham, North Carolina 27708, USA; 3 University Program in Genetics and Genomics, Duke University Medical Center, Durham, North Carolina 27710, USA; 4 Department of Mechanical Engineering, Columbia University, New York, New York 10032, USA; 5 Center for Advanced Genomic Technologies, Duke University, Durham, North Carolina 27710, USA; 6 Nyrada Inc., Gordon, New South Wales, Australia

## Abstract

Atherosclerosis is a primary precursor of cardiovascular disease (CVD), the leading cause of death worldwide. While proprotein convertase subtilisin/kexin 9 (PCSK9) contributes to CVD by degrading low-density lipoprotein receptors (LDLR) and altering lipid metabolism, PCSK9 also influences vascular inflammation, further promoting atherosclerosis. Here, we utilized a vascular microphysiological system to test the effect of PCSK9 activation or repression on the initiation of atherosclerosis and to screen the efficacy of a small molecule PCSK9 inhibitor. We have generated PCSK9 over-expressed (P+) or repressed (P−) human induced pluripotent stem cells (iPSCs) and further differentiated them to smooth muscle cells (viSMCs) or endothelial cells (viECs). Tissue-engineered blood vessels (TEBVs) made from P+ viSMCs and viECs resulted in increased monocyte adhesion compared to the wild type (WT) or P− equivalents when treated with enzyme-modified LDL (eLDL) and TNF-α. We also found significant viEC dysfunction, such as increased secretion of VCAM-1, TNF-α, and IL-6, in P+ viECs treated with eLDL and TNF-α. A small molecule compound, NYX-1492, that was originally designed to block PCSK9 binding with the LDLR was tested in TEBVs to determine its effect on lowering PCSK9-induced inflammation. The compound reduced monocyte adhesion in P+ TEBVs with evidence of lowering secretion of VCAM-1 and TNF-α. These results suggest that PCSK9 inhibition may decrease vascular inflammation in addition to lowering plasma LDL levels, enhancing its anti-atherosclerotic effects, particularly in patients with elevated chronic inflammation.

## INTRODUCTION

Proprotein convertase subtilisin/kexin 9 (PCSK9) plays a crucial role in lipid metabolism through its ability to degrade low-density lipoprotein receptors (LDLR), leading to increases in circulating LDL, and greater incidence of cardiovascular disease (CVD).^1^ Naturally occurring PCSK9 gain-of-function (GOF) mutations increase serum LDL levels^2^ while loss-of-function (LOF) mutations decrease serum LDL levels.^3^ Patients with these LOF mutations showed a substantial reduction in CVD risk.^4^ The progression of atherosclerosis and CVD has long been associated with inflammation.^5,6^ In addition to its effect on circulating LDL levels, PCSK9 has also been linked to inflammation. *In vivo* and *in vitro* studies showed a positive correlation between PCSK9 and pro-inflammatory cytokine expression and secretion, white blood cell count, and monocyte accumulation and activation.^7–12^ PCSK9 knockdown or inhibition has resulted in lower levels of inflammatory cytokines, reduced monocyte adherence at atherosclerotic lesion sites, and decreased macrophage content in lesions.^13–15^ Inhibiting PCSK9 expression or function could impact not only circulating LDL levels but atherosclerosis-promoting inflammation as well.^16^

Several therapies have been developed to reduce PCSK9 levels including monoclonal antibodies,^17–19^ antisense oligonucleotides,^20^ small molecule inhibitors,^21^ CRISPR-based gene editors^22^ and epigenome editors,^23^ and vaccinations.^24^ While many of these show substantial decreases in LDL levels, their impact on inflammation is still unclear. Patient trials conducted using anti-PCSK9 monoclonal antibodies found no improvement in high-sensitivity C-reactive protein (CRP), a marker of systemic inflammation.^25,26^ Furthermore, highly elevated CRP levels predicted increased risk of cardiovascular events, even after significant reductions of LDL cholesterol with PCSK9 inhibitors.^27^ However, *in vivo* mouse studies evaluating PCSK9 inhibition saw reductions in intracellular adhesion molecule 1 (ICAM-1) in plaque endothelial cells (EC), decreased monocyte recruitment and adhesion to the vascular wall, and decreased NF-κB expression in atherosclerotic lesions.^13,14,28^ Additionally, a study in patients with familial hypercholesterolemia found anti-inflammatory changes in monocyte phenotype did not coincide with a decrease in CRP levels.^29^ A recent clinical study investigating the effect of PCSK9 inhibition on inflammation found patients treated with an anti-PCSK9 antibody had lower levels of IL-1β and TNF-α in plaques and increased plaque stability.^16,30^ Further investigation is needed as PCSK9 inhibitors appear to reduce inflammation at the lesion site but have variable effects on markers of systemic inflammation in patients.^13,14,25,26,28^ Our tissue-engineered blood vessels (TEBVs) provide a unique platform for studying the effects of PCSK9 inhibition on the human vascular microenvironment independent of systemic responses and could aid in reconciling some of the discrepancies between animal and clinical studies.

Here, we describe a TEBV model of PCSK9 activation or repression using human induced pluripotent stem cell (iPSC) derived endothelial (viECs) and smooth muscle cells (viSMCs). Our aims are (1) to elucidate the role of PCSK9 in vascular inflammation and initiation of atherosclerosis, and (2) to utilize the TEBV model for drug development by evaluating vascular cell-specific responses to drug treatment. We regulated PCSK9 expression using CRISPR activation or inhibition technology. To generate cells that activated or repressed PCSK9, we employed a ^VP64^dSpCas9^VP64^ or a dSpCas9^KRAB^ construct, respectively.^31,32^ Previously, we have used the TEBV model to emulate early stage atherosclerosis^33^ and correlate the localization of foam cell formation with hydrodynamics at the bifurcated areas of branched vessels.^34^ In this study, we show that PCSK9 activation increases pro-inflammatory marker expression in viSMCs, secretion of adhesion molecules by viECs, and accumulation of monocytes in TEBVs. We tested a small molecule compound, NYX-1492, which was designed to block PCSK9 binding with the LDLR. We found that the small molecule compound reduced inflammation in viSMCs and viECs and decreased monocyte accumulation in TEBVs. Our study shows that PCSK9 is a pro-inflammatory mediator in human vascular cells and that PCSK9 inhibition has the potential to decrease inflammatory risk at the site of atherosclerotic plaque formation.

## RESULTS

### Characterization of PCSK9 activation and repression in vascular cells

To generate cells that activated (P+) or repressed (P−) PCSK9, we employed a ^VP64^dCas9^VP64^ or a dCas9^KRAB^ construct, respectively.^31,32^ Two different iPSC lines were used (DU11 and WTC11) to test single guide RNA (gRNA) efficacy. Cells were transduced with lentivirus containing either the ^VP64^dCas9^VP64^ transcriptional activator or the dCas9^KRAB^ transcriptional repressor with corresponding gRNAs. We used antibiotic selection to purify the successfully transduced iPSC populations and measured PCSK9 expression as compared to wild-type (WT) iPSCs using qRT-PCR. We examined three gRNAs from the PCSK9 activation system (A-gRNA) and four gRNAs from the PCSK9 repressor system (R-gRNA). A-gNRA #3 produced significant PCSK9 activation and R-gRNA #4 repressed PCSK9 (supplementary material Fig. S1). These gRNAs were used in subsequent experiments.

**FIG. 1.**
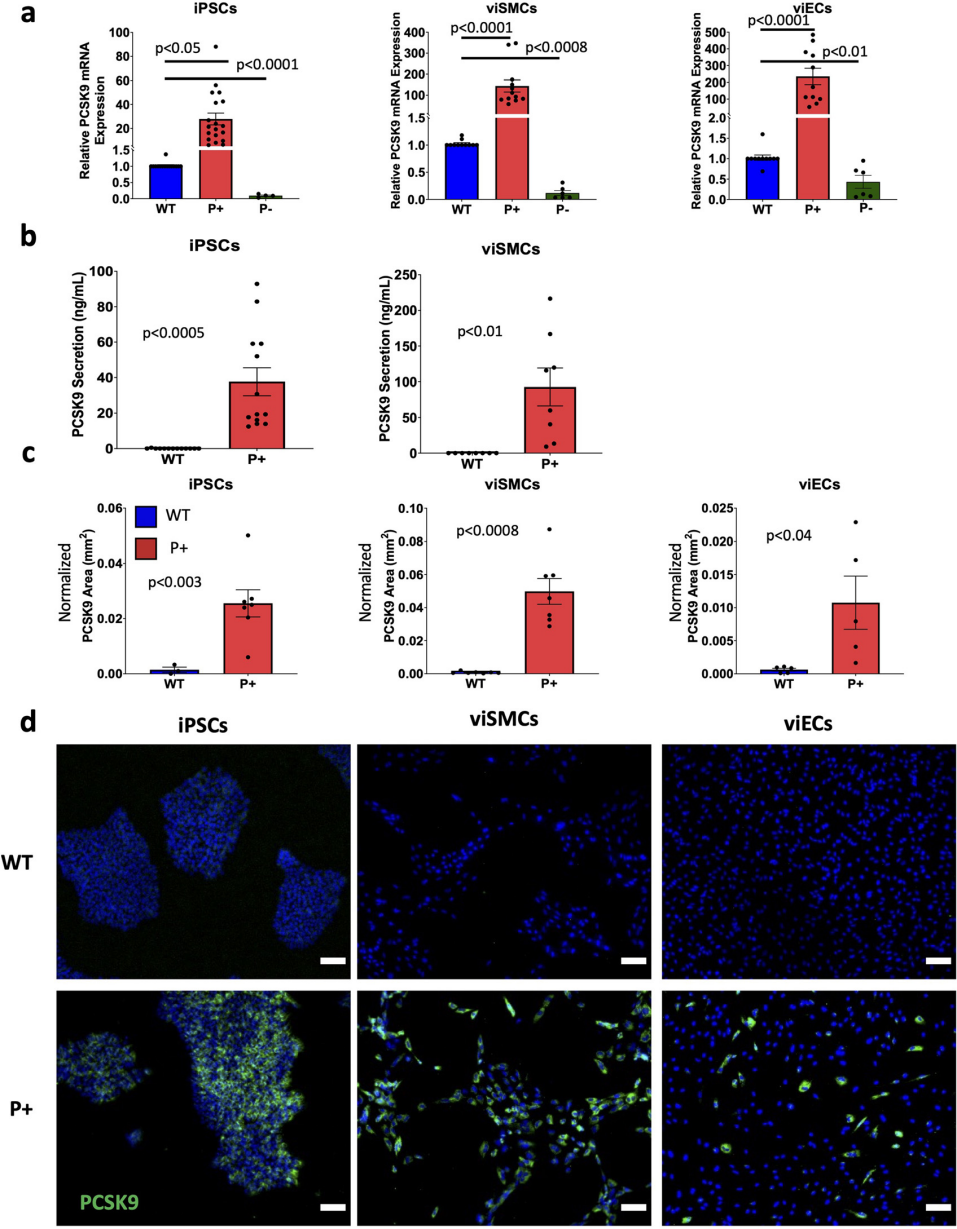
PCSK9 activation (P+) and repression (P−) in iPSCs, iPSC-derived vascular endothelial (viEC), and smooth muscle (viSMC) cells. (a) PCSK9 mRNA expression in wild-type (WT), P+, and P− iPSCs, viSMCs, and viECs. iPSC results from WTC11 and DU11 donors (WT n = 15, P+ n = 19, and P− n = 4). viSMC results from WTC11 and DU11 donors (WT n = 11, P+ n = 12, and P− n = 6). viEC results from WTC11 and DU11 donors (WT n = 11, P+ n = 11, and P− n = 6). Statistical analysis was performed using a one-way ANOVA with post-hoc Tukey test for multiple comparisons for each cell type. (b) PCSK9 secretion from WT and P+ iPSCs and viSMCs. iPSC results are from two donors (WT n = 13, P+ n = 13). viSMC results from WTC11 donor (WT n = 8, P+ n = 8). Statistical analysis was performed using a Student's t-test for each cell type. (c) Quantification of PCSK9 positive area for iPSCs, viSMCs, and viECs from (d). iPSC results from two donors (WT n = 3, P+ n = 7). viSMC results from one donor (WT n = 7, P+ n = 7). viEC results from WTC11 donor (WT n = 5, P+ n = 5). Statistical analysis was performed using a Student's t-test for each cell type. (d) Representative immunofluorescent images of PCSK9 expression in WT and P+ iPSCs, viSMCs, and viECs. Cell area was normalized to cell number. Scale bar = 100 *μ*m.

PCSK9 activation in iPSCs was evaluated by measuring mRNA levels, protein secretion, and protein expression compared to wild-type (WT) iPSCs that were not transduced. Results were pooled from the DU11 and WTC11 cell lines as no differences were found between mRNA levels (P+ p-value = 0.39, P− p-value = 0.52), protein secretion (WT p-value = 0.06, P+ p-value = 0.28), or protein expression (WT p-value = 0.18, P+ p-value = 0.78). We found a significant increase in PCSK9 mRNA, PCSK9 secretion, and PCSK9 area in the P+ iPSCs compared to WT iPSCs [Figs. 1(a)–1(d)]. Similarly, PCSK9 repression (P−) in iPSCs was evaluated by measuring PCSK9 mRNA levels compared to WT iPSCs. We found significantly decreased mRNA levels in the P− iPSCs [Fig. 1(a)].

To further evaluate the effects of PCSK9 on vascular cells, P+, P−, and WT viSMCs and viECs were differentiated from corresponding iPSC variants as reported previously.^35^ Compared to their WT control, PCSK9 mRNA levels were significantly higher in P+ viSMCs and viECs, and significantly lower in P− viSMCs and viECs [Fig. 1(a)]. PCSK9 secretion was significantly higher in P+ iPSCs and viSMCs but was not detectable in P+ viECs [Fig. 1(b)]. Compared to the WT control, the cell area immunostained for PCSK9 was significantly higher in P+ iPSCs, viSMCs, and viECs [Figs. 1(c) and 1(d)]. Given the low levels of PCSK9 protein immunofluorescence on WT cells, PCSK9 protein measurements were not done on P− iPSCs, viSMCs, or viECs.

**FIG. 2.**
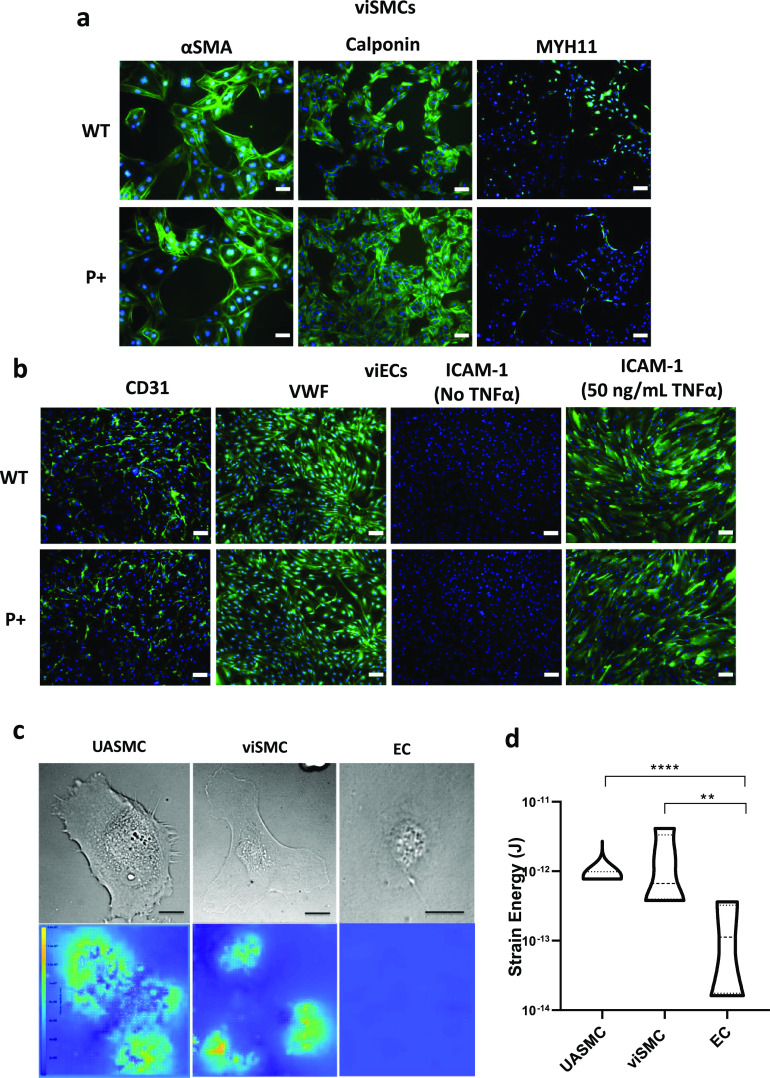
Characterization of viSMCs and viECs modified to activate PCSK9 expression. (a) Representative immunofluorescent images of WT and P+ viSMCs expressing markers of mature smooth muscle: αSMA, calponin, and MYH11. αSMA scale bars = 50 *μ*m. Calponin and MYH11 scale bars = 100 *μ*m. (b) Representative immunofluorescent images of WT and P+ viECs expressing markers of mature and functional endothelial cells: CD31, vWF, and ICAM-1 in the presence or absence of TNFα. Scale bars =100 *μ*m. (c) Representative bright field images UASMC, WT viSMC, and EC on 7 kPa PAA gels (top, scale bars indicate 20 *μ*m) and the associated strain energy visualized on each cell (bottom) by traction force microscopy. (d) Violin plot of strain energy calculated on each cell type, center dashed line indicating median value, N = 32 per cell type from four independent experiments. One-way ANOVA with Tukey post hoc test, ^*^P < 0.05 ^**^P < 0.005, ^***^P < 0.0005, and ^****^ P < 0.0001.

After validating the efficacy of PCSK9 activation and repression in viSMCs and viECs, we sought to ensure the additional gene regulation machinery was not interfering with cell differentiation and maturation. We measured the expression of key smooth muscle or endothelial cell markers in P+ and WT viSMCs and viECs, respectively. P+ viSMCs expressed comparable levels of α-smooth muscle actin (α-SMA), calponin, and myosin heavy chain 11 (MYH11) as WT viSMCs [Fig. 2(a)]. Similarly, P+ viECs expressed similar levels of CD31 and von Willebrand factor (VWF) compared to WT viECs [Fig. 2(b)]. These viECs also demonstrated robust expression of intercellular adhesion molecule-1 (ICAM-1) after exposure to tumor necrosis factor α (TNF-α) [Fig. 2(b)].

In addition to differentiation makers, traction force microscopy (TFM) was used to compare viSMCs' contractile capability with those of human umbilical artery smooth muscle cells (UASMCs) and endothelial cells (ECs) as positive and negative controls, respectively [Figs. 2(c) and 2(d)]. All three cell types adhered and spread well on a collagen-coated polyacrylamide (PAA) gel surface and fluorescent particles embedded in the PAA gel were visualized. Upon treatment with cell-releasing reagents, i.e., Accutase for viSMCs or 0.25% Trypsin for UASMCs or ECs, respectively, cells began detaching from the PAA gel and the fluorescent particles were displaced. Strain energy was calculated from the displacement of these fluorescent particles.^36^ WTC11 viSMCs showed comparable strain energy to that of UASMCs but were approximately ten-fold higher than that of ECs [Fig. 2(d)].

### Characterization of PCSK9 activated vascular cells in TEBVs

**FIG. 3.**
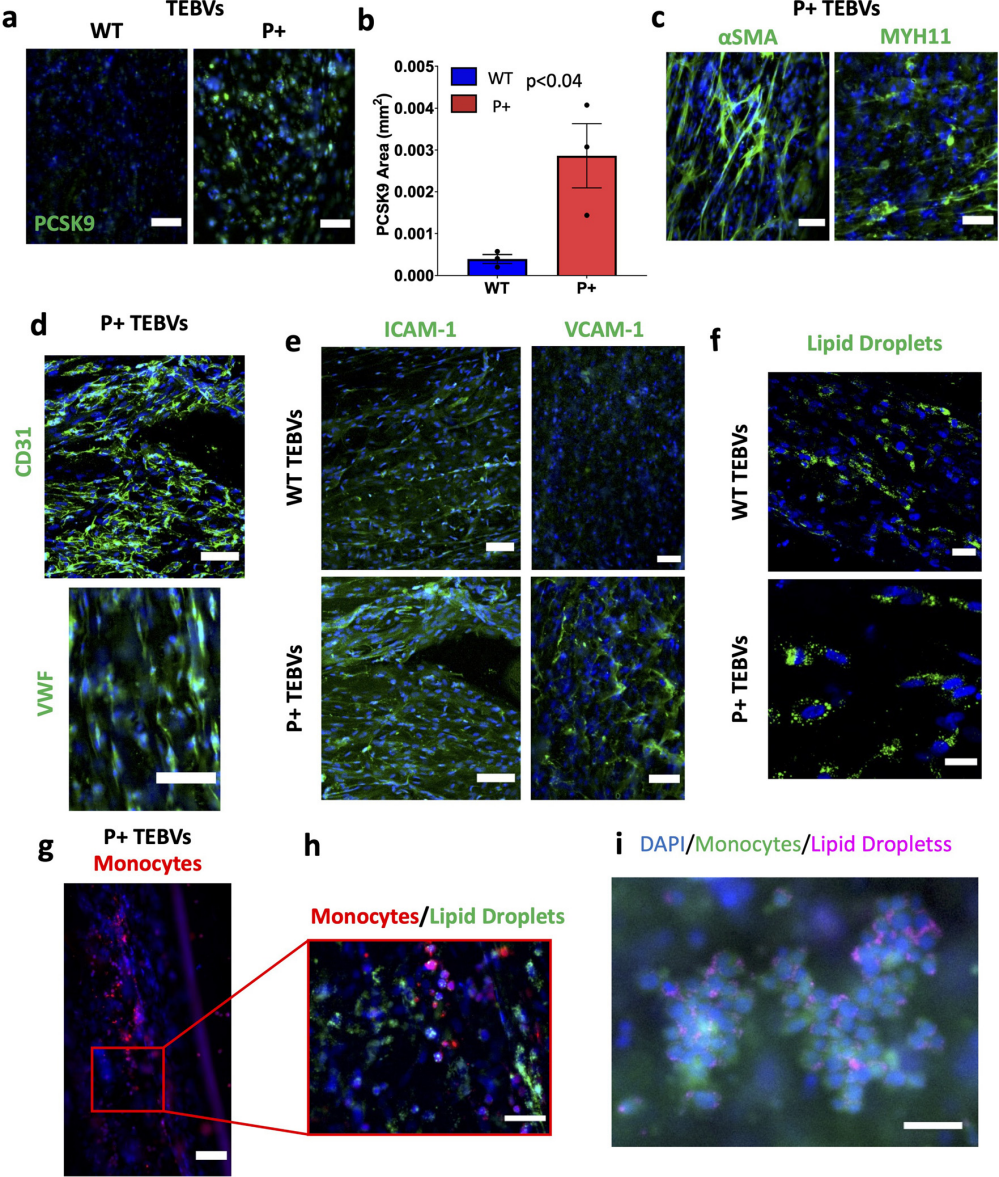
Characterization of TEBVs fabricated with cells modified to activate PCSK9 expression. (a) Representative *en face* immunofluorescent images of PCSK9 expression in WT and P+ TEBVs. Scale bars = 100 *μ*m. (b) Quantification of PCSK9 positive area from (a). Results from WTC11 donor (WT n = 3, P+ n = 3). Statistical analysis was performed using a Student's t-test. (c) Representative immunofluorescent images of P+ TEBVs expressing mature smooth muscle markers, αSMA and MYH11. Scale bars = 100 *μ*m. (d) Representative immunofluorescent images of P+ TEBVs expressing mature endothelial cell markers, CD31 and vWF. Scale bars = 100 *μ*m. (e) Representative immunofluorescent images of WT and P+ TEBVs expressing adhesion molecules ICAM-1 and VCAM-1 after exposure to 50 ng/ml TNF-α and 60 *μ*g/ml eLDL. Scale bars = 100 *μ*m. (f) Representative immunofluorescent images of lipid accumulation in the smooth muscle cells of the WT and P+ TEBVs after exposure to 60 *μ*g/ml eLDL. Scale bar = 25 *μ*m. (g) Representative immunofluorescent images of monocyte adhesion in P+ TEBVs after exposure to 50 ng/ml TNF-α and 60 *μ*g/ml eLDL. Scale bar = 100 *μ*m. (h) Magnification of specific area in the image from (g) showing monocyte adhesion and lipid accumulation in smooth muscle cells and monocytes. Scale bar = 50 *μ*m. (i) Lipid droplets (purple) shown around monocytes (green) in TEBV. Scale bar = 50 *μ*m.

Previously, we have established a TEBV model to study early stage atherosclerosis *in vitro* both in linear^33^ and branched^34^ formats. To study the effects of PCSK9 activation and repression on atherosclerosis progression in the vascular microenvironment, we fabricated TEBVs using WT, P+, and P− viSMCs and viECs. Flow rates that favor the promotion of atherosclerosis were used.^33,37^ PCSK9 expression and markers of SMCs and ECs were measured in P+ TEBVs after five to seven days of perfusion. A significant increase in PCSK9 area was found in P+ TEBVs compared to WT [Figs. 3(a) and 3(b)]. P+ TEBVs also demonstrated a robust expression of αSMA, MYH11, CD31, and vWF, indicative of mature SMCs and ECs [Figs. 3(c) and 3(d)]. We then assessed relevant functional measures of P+ TEBVs by treating them with TNF-α and an enzymatically modified LDL, eLDL^38^ for 48 h and evaluating adhesion molecule expression and lipid accumulation, as we have done previously.^33^ We chose to treat TEBVs with eLDL over unmodified LDL or commonly used oxidized LDL (oxLDL) because eLDL treatment more readily induces foam cell formation,^38^ better simulating a more advanced atherosclerotic environment (supplementary material Fig. S2). P+ TEBVs showed equal or greater expression of ICAM-1 and vascular cell adhesion molecule 1 (VCAM-1) compared to WT TEBVs [Fig. 3(e)]. They also showed comparable levels of lipid droplet accumulation [Fig. 3(f)]. The TEBVs were perfused with labeled monocytes during TNF-α and eLDL treatment to evaluate adhesion of these monocytes to the cell wall, a hallmark of early stage atherosclerosis.^39,40^ Monocytes adhered along the lumen of TEBVs, with some accumulating lipid droplets, suggesting early foam cell formation^41^ [Figs. 3(g)–3(i)].

### PCSK9 activation promotes early-stage atherosclerosis in TEBVs

**FIG. 4.**
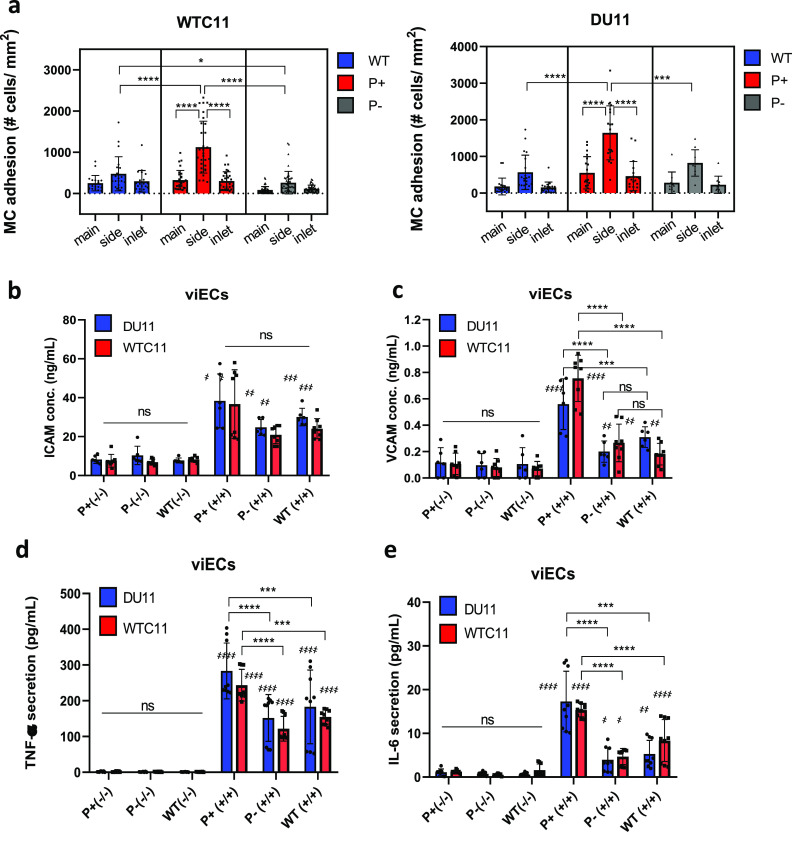
Effect of PCKS9 variants in early atherosclerosis in TEBVs. (a) Monocyte adhesion on inlets, main, and side outlets of branched TEBVs from WTC11 and DU11 patients' P+, P−, and WT cells after treatment with 10 *μ*g/ml eLDL and 50 ng/ml TNF-α for 96 h. n = 30 images/location, 3–4 vessels pooled from three independent experiments. One-way ANOVA with Tukey post hoc test, ^*^P < 0.05, ^**^P < 0.005, ^***^P < 0.0005, and ^****^P < 0.0001. (b) Secreted ICAM-1 and (c) VCAM-1 after treatment with 10 *μ*g/ml eLDL and 50 ng/ml TNF-α (+/+) or non-treated (−−). n = 8, data from four independent experiments, one-way ANOVA with Tukey post hoc test. (d) Secreted TNF-α and (e) IL-6 after treatment with 10 *μ*g/ml eLDL and 50 ng/ml TNF-α (+/+) or non-treated (−/−). n = 9, data from three independent experiments, one-way ANOVA with Tukey post hoc test, ^***^P < 0.0005, ^****^P < 0.0001 compared between variants. P < 0.05, P < 0.005, and P < 0.0005 compared to their non-treated (−/−) counterparts.

For both iPS cell lines, WTC11 and DU11, branched TEBVs made from P+ viSMCs and viECs resulted in significantly higher monocyte adhesion in their side outlets compared to those made from either WT or P− viSMCs and viECs [Fig. 4(a)]. In contrast, P− TEBVs did not show significant differences in monocyte adhesion compared to WT TEBVs [Fig. 4(a)]. No notable difference was found in monocyte adhesion between TEBVs of WTC11 and DU11 patients' cells in any condition. The inlet and main outlet areas had fewer adhered monocytes compared to the side outlet area of all branched TEBVs, likely due to local hydrodynamics.^34^

To find a plausible cause of the promoted monocyte adhesion in P+ TEBVs, we studied the secretion of surface adhesion molecules and pro-inflammatory cytokines of viECs. Secretion levels from viECs for both ICAM-1 and VCAM-1 were significantly increased by treatment of eLDL and TNF-α (+/+) [Figs. 4(b) and 4(c)], compared to the non-treated condition (−/−). Moreover, VCAM-1 secretion by P+ viECs was significantly higher compared to P− or WT viECs of both patients [Fig. 4(c)]; however, ICAM-1 secretion was not affected by PCSK9 variants [Fig. 4(b)]. Similarly, pro-inflammatory cytokines, TNF-α and IL-6, were secreted significantly more by P+ viECs than WT or P− variants when treated with eLDL and TNF-α (+/+) [Figs. 4(d) and 4(e)]. TNF-α and IL-6 secretion by viSMCs was much lower than by viECs and was not affected by PCSK9 variants (supplementary material Fig. S3). These results implied that monocyte adhesion was promoted by PCSK9 activation in inflammatory conditions and was caused by increased VCAM-1 secretion and increased pro-inflammatory cytokine secretion in P+ viECs.

### Small molecule PCSK9 inhibitor attenuates inflammation in vascular cells and TEBVs

While injectable anti-PCSK9 monoclonal antibodies have shown benefits in lowering plasma LDL levels and reducing cardiovascular events in patients,^18,19^ their high cost and route of administration limit widespread use. Small molecule PCSK9 inhibitors offer a potentially inexpensive, oral alternative to monoclonal antibody therapy. Nyrada Inc. recently published a series of small molecule compounds, targeting the PCSK9-LDLR binding site.^42,43^ One compound, NYX-1492, exhibited a strong *in vivo* pharmacokinetic profile following oral and IV administration, with a high maximum serum concentration and relatively long half-life (supplementary material Fig. S4). These characteristics make it a promising candidate for further drug testing. We explored the utility of both branched and non-branched TEBV models to investigate the potential anti-inflammatory effects of PCSK9 inhibition, by NYX-1492 on the vascular microenvironment. The chemical structure and NMR data of NYX-1492 are available in supplementary material Fig. S5(a).

**FIG. 5.**
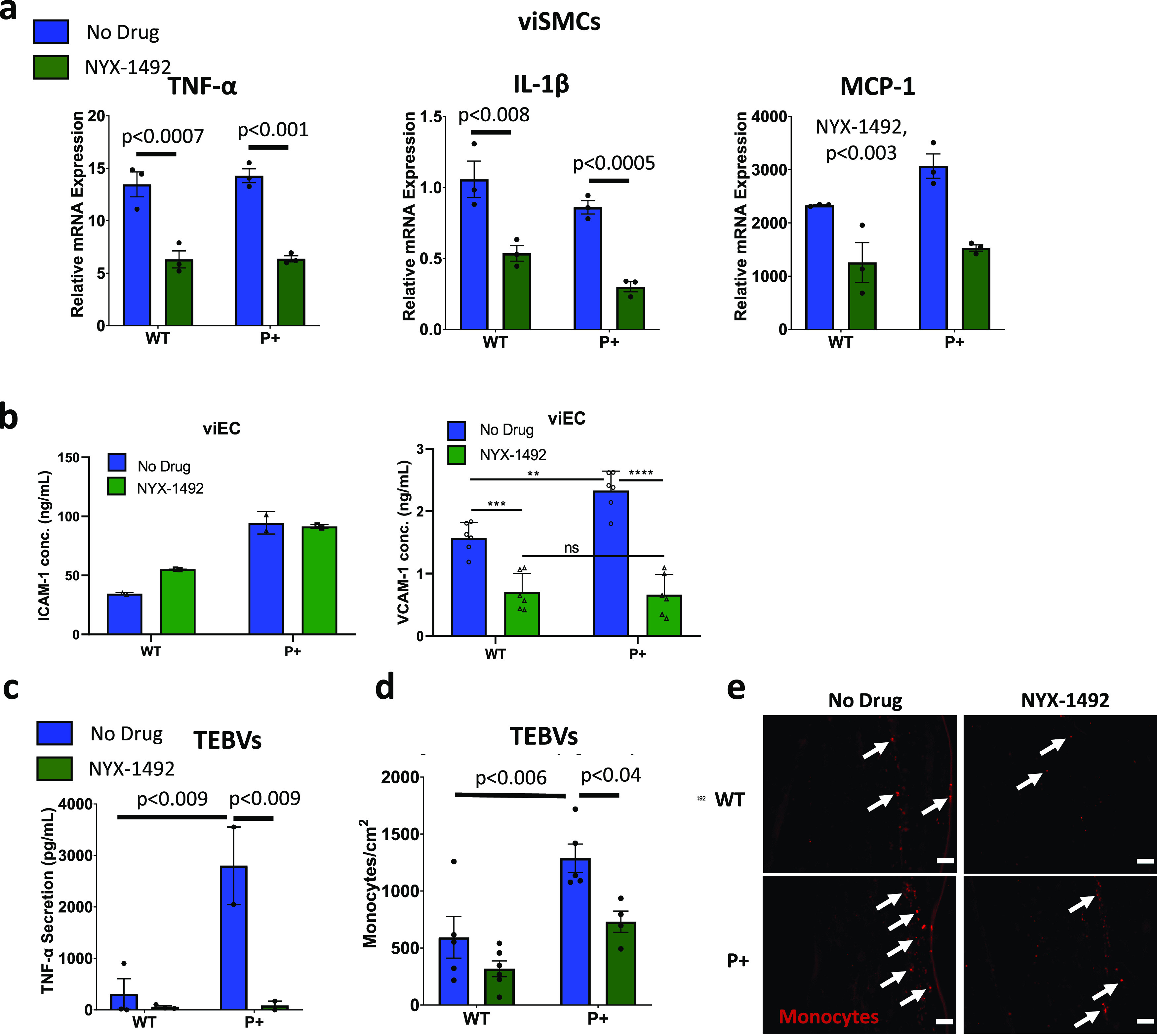
Small molecule PSCK9 inhibitors reduce inflammation and monocyte adhesion in P+ TEBVs. (a) mRNA expression of inflammatory cytokines, TNF-α, IL-1β, and MCP-1 in WTC11 WT and P+ viSMCs after 48 h of exposure to 50 ng/ml TNF-α and 10 *μ*g/ml eLDL, with or without 8 *μ*M NYX-1492. Results from WTC11 donor (WT No Drug n = 3, WT NYX-1492 n = 3, P+ No Drug n = 3, and P+ NYX-1492 n = 3). Statistical analysis was performed using a two-way ANOVA with post-hoc Tukey test for multiple comparisons for each cytokine. (b) ICAM-1 and VCAM-1 secretion from DU11 WT and P+ viECs after 48 h of exposure to 50 ng/ml TNF-α and 10 *μ*g/ml eLDL, with or without 8 *μ*M NYX-1492. n = 6 from two experiments, experiments were repeated twice, one-way ANOVA with post-hoc Tukey test, ^*^p < 0.05, ^**^p < 0.01, ^***^p < 0.001, or ^****^p < 0.0001. (c) TNF-α secretion from WTC11 WT and P+ non-branched TEBVs after exposure to 50 ng/ml TNF-α and 60 *μ*g/ml eLDL for 96 h, with or without 8 *μ*M NYX-1492. The medium was replaced at around 84-h timepoint to remove exogenous TNFα 12 h prior to collection for analysis. Results from WTC11 donor (WT No Drug n = 3, WT NYX-1492 n = 3, P+ No Drug n = 2, and P+ NYX-1492 n = 2). Statistical analysis was performed using a two-way ANOVA with post-hoc Tukey test for multiple comparisons for each cytokine. (d) Quantification of U937 monocyte adhesion in (e). Results from WTC11 donor (WT No Drug n = 5, WT NYX-1492 n = 6, P+ No Drug n = 5, and P+ NYX-1492 n = 4). Statistical analysis was performed using a two-way ANOVA with post-hoc Tukey test for multiple comparisons for each cytokine. (e) Representative immunofluorescent images of U937 monocyte adhesion in WTC11 WT and P+ non-branched TEBVs after exposure to 50 ng/ml TNF-α and 60 *μ*g/ml eLDL for 96 h, with or without 8 *μ*M NYX-1492. Arrows indicate areas with adhered monocytes. Scale bars = 100 *μ*m.

viSMCs and viECs were treated with 50 ng/ml TNF-α and 10 *μ*g/ml eLDL with or without NYX-1492 for 48 h. We found a significant reduction in mRNA expression of inflammatory molecules TNF-α, IL-1β, and MCP-1 in both P+ and WT viSMCs [Fig. 5(a)]. VCAM-1 secretion significantly decreased after NYX-1492 treatment for both WT and P+ viECs, but ICAM-1 secretion was not affected [Fig. 5(b)]. TNF-α and IL-6 secretion in viECs was not significantly affected by 48-h treatment of NYX-1492 (supplementary material Fig. S6). We then fabricated P+ TEBVs and treated them with TNF-α and eLDL for 96 h, with or without NYX-1492 [Figs. 5(c)–5(e) for non-branched TEBVs, and supplementary material Fig. S7 for branched TEBVs]. TNF-α secretion was significantly reduced in P+ TEBVs, but not WT TEBVs, that were treated with NYX-1492 [Fig. 5(c)]. Monocytes were perfused through the TEBVs at 1 × 10^6^ cells/ml for the final 48 h of TNF-α and eLDL treatment. P+ TEBVs treated with TNF-α and eLDL have significantly greater monocyte adhesion than WT TEBVs [Figs. 5(d) and 5(e)]. Monocyte adhesion was also significantly decreased in P+ TEBVs treated with NYX-1492 but not in WT TEBVs [Figs. 5(d) and 5(e) and supplementary material Fig. S7].

## DISCUSSION

In this work, we developed a model of PCSK9 activation in combination with a three-dimensional blood vessel model of early-stage atherosclerosis. We found that PCSK9 activation increased vascular inflammation and promoted atherogenesis in the TEBV models. Our *in vitro* model allowed us to separately examine the effects of inflammation and modified LDL on atherogenesis by exposing the TEBVs to TNF-α, eLDL, or a combination of the two. We then evaluated the influence of PCSK9 activation on vascular health in these different atheroprone environments. In addition, we assessed the impact of TNF-α and eLDL treatment on viECs and viSMCs individually, with and without PCSK9 activation, to determine how each contributed to the inflammatory response within the vascular microenvironment.

We generated vascular ECs and SMCs from iPSCs, allowing for easier and more consistent genome modification using CRISPR activation and repression technology. PCSK9 activated cells showed robust increases in PCSK9 expression at the gene and protein levels. This modification had no apparent detrimental effects on viEC or viSMC differentiation and maturation. viECs expressed common EC markers, CD31 and VWF, and responded to TNF-α, through a robust expression of ICAM-1, while viSMCs expressed common SMC markers, αSMA and calponin. There were low levels of MYH11 expression in viSMCs, but this is similar to primary SMCs when cultured *in vitro*.^44^ SMC differentiation was also confirmed from the contractility of viSMCs, which was comparable to that of UASMCs as measured by TFM. Additionally, MYH11 expression increased when viSMCs were cultured with viECs in the 3D, dynamic environment of a TEBV, suggesting the more physiological conditions may lead to a more contractile phenotype.^45^ TEBVs also expressed the common EC markers, CD31 and vWF and showed robust ICAM-1 and VCAM-1 expression when stimulated with TNF-α, indicating a functional, intact endothelium. Previously, we showed foam cell formation in fibroblasts,^33^ directly reprogrammed iSMCs,^34^ and macrophages within a TEBV model of atherosclerosis.^33^ Here, we observe robust lipid accumulation in viSMCs and adhered monocytes within the TEBVs. This indicates the PCSK9 activation model also exhibits some of the hallmark features of atherosclerosis, allowing for investigation of how PCSK9 might affect these disease characteristics.

We investigated the impact of PCSK9 activation on inflammation in a TEBV model of atherosclerosis. PCSK9 activation resulted in higher monocyte adhesion in the side outlet of branched TEBVs, where flow characteristics mimic those found at branched sites of arteries *in vivo*.^34^ These areas of low, oscillatory shear stress have been shown to locally increase PCSK9 levels, elevate inflammatory molecule expression, and promote monocyte adhesion,^40,46^ similar to our TEBV model. Monocyte transmigration in TEBVs occurs in response to TNF-α^47^ or eLDL^33^ and the process from attachment to appearance in the subendothelial space takes about 5–7 min.^47^ Furthermore, PCSK9 activation promotes macrophage activation, leading to their increased accumulation at lesion sites.^12^ While we did not directly measure macrophage activation, this may have contributed to the increase in adhesion we saw in P+ TEBVs compared to WT. In addition, the higher VCAM-1, TNF-α, and IL-6 secretion by P+ viECs may have contributed to the increased monocyte adhesion seen in the P+ TEBVs. Previous reports have shown that ECs exposed to extracellular vesicles from PCSK9-overexpressing SMCs had increased VCAM-1 and IL-6 expression.^48^ Similarly, knocking down PCSK9 using siRNA showed reduced VCAM-1 expression in human umbilical artery endothelial cells.^49^ Our results corroborate these previous findings and suggest that PCSK9 activation can lead to EC activation, contributing to increased monocyte adhesion. A future study could perform proteomics analysis on the TEBVs to obtain a more comprehensive understanding of the inflammatory landscape within the vasculature resulting from PCSK9 activation.

Many therapies have been developed to inhibit PCSK9 to impart the benefits seen in individuals with natural LOF mutations.^18–22,24^ While these therapies can demonstrate reductions in circulating LDL, their impact on the LDLR-independent effects of PCSK9 remain poorly understood.^8,9^ We hypothesized that inhibiting PCSK9 could reduce local inflammation in the vascular microenvironment. To control for any confounding effects resulting from decreased uptake of LDL due to PCSK9 inhibition, we treated our samples with eLDL. Rather than being taken up by the LDLRs, eLDL uptake occurs via other mechanisms such as macropinocytosis^38^ or the S100/RAGE-mediated pathway;^50^ PCSK9 inhibition is not known to affect these pathways. We found significant decreases in the inflammatory response in viSMCs, viECs, and TEBVs treated with NYX-1492. The reduction in monocyte adhesion seen in the NYX-1492-treated P+ TEBVs could be due, in part, to the significant decrease in VCAM-1 expression we saw in treated P+ viECs. VCAM-1 is known to promote monocyte adhesion in atherosclerotic lesions^51^ and previous studies have shown PCSK9 inhibition can reduce VCAM-1 expression in ECs.^49^ These results highlight the utility of the TEBV system for testing new therapeutics and investigating biological mechanisms. Interestingly, we found decreases in inflammatory marker expression in both WT and P+ viSMCs and viECs that were treated with NYX-1492. While WT cells do express low levels of PCSK9 that could be affected by treatment with the inhibitor, these levels are negligible compared to the P+ cells. We would therefore expect any anti-inflammatory response from PCSK9 inhibition to be much greater in the P+ cells. This differential response between WT and P+ samples was seen in the TEBV results, with only P+ TEBVs having significant reductions in TNF-α secretion and monocyte adhesion. It is possible that the NYX-1492 compound has some inherent anti-inflammatory properties that are independent of blocking PCSK9 binding. Additional studies will be needed to elucidate the mechanism behind this potential benefit.

We have demonstrated that PCSK9 activation can exacerbate the local inflammatory response in vascular cells in an *in vitro* model of early-stage atherosclerosis. One factor influencing this inflammatory response could be the PCSK9 concentration itself. While significantly higher than WT cells, PCSK9 overexpressing cells secreted relatively low amounts of PCSK9 compared to concentrations found in the patient's circulation.^52–54^ This may explain some of the subtle or inconsistent changes we saw in inflammatory marker expression and secretion in the P+ vascular cells, as well as why we did not observe a proinflammatory response in P+ cells that were not treated with TNF-α (supplementary material Fig. S8). In observational studies, increasing serum PCSK9 levels were linearly associated with increased plaque fraction of necrotic core tissue.^55^ Refining our *in vitro* model to produce more PCSK9 could provide insight into the relationship between increasing PCSK9 and increasing vascular inflammation. In addition to the inflammation caused by local endothelial and smooth muscle cells, circulating monocytes and macrophages can also contribute to the inflammatory response through cytokine secretion.^9^ An abundance of proinflammatory monocytes and macrophages are found in the atherosclerotic plaque,^9,56^ and studies show they can be activated by PCSK9.^12^ These activated macrophages secrete proinflammatory cytokines, exacerbating the inflammatory response of local vascular cells.^9,10^ PCSK9 may promote additional cytokine secretion from monocytes and macrophages. A future study could evaluate monocyte inflammation in response to PCSK9 activation and PCSK9 repression using iPSC-derived monocytes. This future study could also take advantage of the utility of the TEBV system by mixing the various cell types (viECs, viSMCs, and monocytes) with differing levels of PCSK9 expression (P+, P−, or WT) to further elucidate their individual contributions to the overall inflammatory response.

### Conclusion

In this study, we explored the utility of the established TEBV platforms to study the effect of PCSK9 activation or repression on vascular inflammation and the initiation of atherosclerosis. Furthermore, we leveraged the TEBV model to screen a small molecule PCSK9 inhibitor as a potential new drug candidate. P+ TEBVs showed higher monocyte adhesion and TNF-α and VCAM-1 secretion, suggesting PCSK9 promotes an atheroprone environment in the vasculature. These results were supported by evidence of increased viEC dysfunction in P+ viECs and higher proinflammatory protein expression and secretion in P+ viSMCs. This indicates the atheroprone characteristics may be due to elevated inflammatory conditions. Our testing of a small molecule PCSK9 inhibitor in pro-atherosclerotic TEBVs revealed that restricting PCSK9 binding can abate its pro-inflammatory effects. This work suggests that PCSK9 may contribute to atherosclerosis beyond lipid metabolism.

## METHODS

### Cell culture

HEK293T cells (American Tissue Collection Center) were cultured in DMEM/F12 supplemented with 10% fetal bovine serum (FBS) and 1% penicillin–streptomycin.

The iPSC line DU11 was reprogrammed episomally from a human foreskin fibroblast line generated from a young postnatal male (line BJ, ATCC CRL-2522). The iPSC line WTC11 was obtained from Coriell Institute and was produced by episomal reprogramming of fibroblasts of a healthy male. Since human cells were obtained from commercial sources, the Duke University Institutional Review Board determined that their use was exempt. iPSCs were maintained in feeder-free conditions on hESC-qualified Matrigel (BD Biosciences) in mTeSR1 medium (STEMCELL Technologies). For routine culture, iPSC colonies were passaged at 80%–90% confluency with 0.5 mM EDTA (Invitrogen). For differentiation, iPSCs were passaged at 80%–90% confluency using Accutase (STEMCELL Technologies) and 10 *μ*M Rock inhibitor Y-27632 (Tocris Bioscience).

The human monocytic cell line U937 was obtained from Sigma-Aldrich. U937 cells were maintained in suspension in RPMI-1640 medium (Sigma-Aldrich) with 10% FBS (Hyclone), 1% GlutaMAX (Gibco), and 1% Antibiotic-Antimycotic (Thermo Fisher Scientific).

THP-1 human monocytes (ATCC) were maintained in RPMI 1640 medium supplemented with 10% FBS, 1% P/S, and β-mercaptoethanol and were passaged every 3–4 days to maintain a cell density of 2–8 × 10^5^ cells/ml.

Human endothelial progenitor cells (EPCs) were derived from human umbilical cord blood and were cultured in T75 cell culture flasks in complete EBM-2 medium with EGM-2 supplement, 10% FBS, and 1% P/S. ECs were obtained by maintaining EPCs in the above EGM-2 supplemented medium for 2–3 passages.

Human aortic smooth muscle cells (Lonza) were cultured using SmGM^TM^-2 Smooth Muscle Cell Growth Medium-2 BulletKit^TM^ (Lonza) with 1% Antibiotic-Antimycotic (Thermo Fisher Scientific).

### viSMC and viEC differentiations

viSMCs and viECs were differentiated using a modified protocol as previously described.^35,57^ iPSCs were passaged using Accutase and seeded onto a Matrigel-coated TCP at 23 000–27 000 cells/cm^2^ (day 0). The following day (day 1), the medium was changed to N2B27 supplemented with 8 *μ*M CHIR99021 (Cayman Chemical) and 25 ng/ml human BMP4 (PeproTech). The medium was not changed again until day 4.

For viSMC differentiation, on day 4, cells were treated with N2B27 medium supplemented with 2 ng/ml Activin A and 10 ng/ml PDGF-ββ (PeproTech). Fresh medium of the same formulation was provided on day 5. On day 6, viSMCs were dissociated using Accutase and seeded onto collagen-coated TCP at densities of 50,000 cells/cm^2^. viSMCs were maintained on TCP coated with 5 *μ*g/ml rat tail collagen I (BD Biosciences) in a one-to-one mix of Neurobasal medium (Thermo Fisher Scientific) and DMEM/F-12 with HEPES (Thermo Fisher Scientific), supplemented with N2 supplement (Thermo Fisher Scientific), B27 supplement without vitamin A (Thermo Fisher Scientific), and treated with 1% P/S. The medium also included 2 *μ*g/ml Heparin (Sigma-Aldrich) and 2 ng/ml Activin A (PeproTech). Fresh 2-mercaptoethanol (Thermo Fisher Scientific) was added to the medium at a 1:1000 dilution, and the medium was changed every other day. Cells were passaged at 80%–90% confluency, and viSMCs at passages 1–4 were used for experiments.

For viEC differentiations, on day 4, cells were treated with StemPro-34 SFM supplemented with 200 ng/ml VEGF165 and 2 *μ*M forskolin (Sigma-Aldrich). On day 5, the conditioned medium was collected and replaced using the same formulation from day 4. This was repeated on day 6. On day 7, the conditioned medium was collected again. All conditioned medium from days 5, 6, and 7 were combined with StemPro-34 SFM (1:1 ratio) with 0.5% P/S and 2 *μ*g/ml Heparin for the initial culture of differentiated viECs. Cells were dissociated with Accutase and washed with MACS buffer containing PBS (Thermo Fisher Scientific) with 0.5% BSA (Sigma-Aldrich-Aldrich) and 2 mM EDTA (Thermo Fisher Scientific). Cells were counted, centrifuged, and resuspended in MACS buffer at a ratio of 80 *μ*l/10^6^ cells. They were then incubated with FcR blocking reagent (Miltenyi Biotec), CD31 magnetic microbeads (Miltenyi Biotec), and CD144 magnetic microbeads (Miltenyi Biotec), at a ratio of 2 *μ*l/10^6^ cells, for 15 min on ice. Cells were washed and then sorted using the MACS column system (Miltenyi Biotec). Sorted cells were seeded on collagen-coated TCP at densities of 40 000–50 000 cells/cm^2^.

viECs were maintained on tissue culture plastic (TCP) coated with 5 *μ*g/ml rat tail collagen I (BD Biosciences) in StemPro-34 SFM (Thermo Fisher Scientific) supplemented with 1% penicillin-streptomycin (P/S, Thermo Fisher Scientific), 1% GlutaMAX (Gibco), 10% heat-inactivated FBS (Thermo Fisher Scientific), 2 *μ*g/ml Heparin (Sigma-Aldrich), and 50 ng/ml VEGF165 (PeproTech). The culture medium was also supplemented with 10 *μ*M of fresh TGFβ pathway inhibitor SB431542 (Sigma-Aldrich)^58,59^ and changed every other day. Cells were passaged at 80%–90% confluency, and viECs at passages 1–3 were used for experiments.

### Generation of plasmid constructs and lentiviral transduction

PCSK9 promoter-targeting gRNA sequences were sourced from previous publications for optimal gene activation^60^ and repression^61^ (with 5' additional nucleotide to increase protospacer length from 19 to 20 bp). For gene activation, PCSK9 gRNAs were cloned into an all-in-one lentiviral vector pLV-hU6-gRNA-hUBC-VP64dCas9VP64-T2A-BlastR (Addgene #66707 in which GFP was replaced with BlastR). For gene repression, PCSK9 gRNAs were cloned into an all-in-one lentiviral vector pLV-hU6-gRNA- hUBC-dCas9KRAB-T2A-PuroR (Addgene #71236).

Lentivirus was produced using HEK293T cells were cultured in Dulbecco's Modified Eagle's Medium supplemented with 10% FBS and 1% penicillin/streptomycin. Approximately 3.5 × 10^6^ cells were plated per 10 cm TCPS dish. 24 h later, the cells were transfected using the calcium phosphate precipitation method with pMD2.G (Addgene #12259) and psPAX2 (Addgene #12260) second generation envelope and packaging plasmids. The medium was exchanged 12 h post-transfection, and the viral supernatant was harvested 24 and 48 h after this medium change. The viral supernatant was pooled and centrifuged at 500 g for 5 min, passed through a 0.45 *μ*m filter, and concentrated to 20× using Lenti-X Concentrator (Clontech) in accordance with the manufacturer's protocol.

For transduction, concentrated viral supernatant was diluted 1–20 in the culture medium of the cells being transduced. Culture medium was changed the day after transduction. A dose of 1 *μ*g/ml puromycin (InvivoGen) for dCas9^KRAB^ or 2 *μ*g/ml blasticidin (InvivoGen) for ^VP64^dCas9^VP64^ was used for selection approximately 72 h after transduction.

### Guide RNA testing

For the dCas9^KRAB^ repressor, we designed and tested five gRNAs (R-gRNAs 1–5) targeting the DNase I hypersensitivity site surrounding the transcriptional start site in PCSK9. For the ^VP64^dCas9^VP64^ activator, we tested three guide RNAs (A-gRNAs 1–3) designed by the Feng Zhang lab that target the first 200 bps upstream of the transcriptional start site of PCSK9. Each dCas9 construct and respective gRNA was delivered into DU11 iPSCs (Duke iPSC Shared Resource Facility) using lentivirus. Transduced cells were selected for using puromycin (dCas9^KRAB^) or blasticidin (^VP64^dCas9^VP64^) antibiotic resistance. RNA was isolated from DU11 iPSCs after seven days of antibiotic selection, and repression or activation was quantified using qRT-PCR of PCSK9 expression normalized to GAPDH. The gRNA that resulted in the greatest reduction in PCSK9 expression was chosen for future knockdown studies, and the one that resulted in the greatest increase in PCSK9 expression was chosen for future upregulation studies.

### Traction force microscopy (TFM)

TFM was used to compare iPSC-derived viSMCs' contractile capability with those of UASMCs and ECs as positive and negative controls, respectively. Strain energy was calculated^36,62^ from the displacement of fluorescent beads embedded in the polyacrylamide (PAA) gels on which cells were seeded and contracted upon treatment of cell-releasing reagents (Accutase for viSMCs, and Trypsin for UASMC or ECs, respectively). PAA gels were prepared following established methods^63^ with slight modification. Briefly, a thin, known stiffness of PAA gel, mixed with fluorescent beads of 200 nm in size (Fluorosphere, Thermo Fisher Scientific), was formed on clean, functionalized (i.e., silanized and then hydroformylated) glass coverslips, and then collagen gel was coated on top of the PAA gel following surface activation by sulphosuccinimidyl-6-(4-azido-2-nitrophenylamino) hexanoate (Sulfo-SANPAH).^64^

### Nanoindentation

Spherical nanoindentation (Piuma, Optics11Life, Amsterdam, NL) was utilized to determine the stiffness of the PAA gels (supplementary material Fig. S9). Prior to testing, samples were swelled overnight in Opti-free contact lens solution (Alcon, Fort Worth, TX, USA) to reduce adhesion between the glass probe and sample.^65^ Using an indenter with a probe radius of 109 *μ*m and cantilever stiffness of 0.53 N/m, samples were indented to a fixed depth of 4 *μ*m for 15 s, corresponding to a 4% strain, to achieve a load relaxation curve approaching equilibrium. A total of 25 indentation points centered on the hydrogel were measured for each sample group; the distance between indentation points was fixed at 200 *μ*m. To determine the equilibrium stiffness (E_∞_) of the hydrogels, load relaxation curves were fitted in MATLAB using an established viscoelastic model.^66,67^ The average Measured Young's modulus, 7 kPa, was used to calculate the strain energy value in TFM analysis. A fair batch-to-batch reproducibility of the gels was observed, and a negligible degree of inter-sample and intra-sample variability was confirmed. Neither the inclusion of fluorescent polystyrene beads nor collagen coating has an appreciable effect on the measured stiffness of PAA gels.

### 2D immunofluorescence staining

iPSCs were grown on Matrigel-coated plates and viECs and viSMCs were grown on collagen-coated plates. Cells were cultured until an appropriate density was obtained, and then they were fixed in 4% paraformaldehyde (Alfa Aesar) for 20 min. Cells were then permeabilized for 10 min in 0.1% TritonX-100 (Sigma-Aldrich) and blocked for at least 1 h at room temperature using a solution of 10% goat serum (Thermo Fisher Scientific) and 5% bovine serum albumin (Sigma-Aldrich). Following blocking, cells were incubated in primary antibodies overnight at 4 °C. The following primary antibodies were used for cell characterization: PCSK9 (Abcam—ab181142, 1:100), αSMA (Abcam—ab5694, 1:100), calponin (Abcam—ab46794, 1:100), MYH11 (Santa Cruz Biotechnology—sc-6956, 1:100), CD31 (Abcam—ab28364, 1:100), VWF (Abcam—ab778, 1:100), and ICAM-1 (Santa Cruz Biotechnology—sc-8439, 1:100). After overnight incubation in primary antibody, cells were incubated for 1 h at room temperature in a solution containing Hoechst dye (nuclear stain—Thermo Fisher Scientific, 1:500) and appropriate fluorescently labeled secondary antibodies: 488-goat anti-mouse IgG (Invitrogen, 1:200) or 488-goat anti-rabbit IgG (Invitrogen, 1:200). Images were acquired using either a Nikon model TE2000-U fluorescence microscope or Leica SP5 inverted confocal microscope.

### Oil Red O Staining

Human aortic smooth muscle cells (AoSMCs) were treated with 50 *μ*g/ml oxLDL or 50 *μ*g/ml eLDL for 48 h and then fixed in 4% paraformaldehyde for 20 min. Following fixation, cells were stained with Oil Red O for detection of foam cell formation as previously described.^68^ Briefly, fixed cells were incubated in 60% isopropanol for 15 s, followed by an incubation in Oil Red O for 5 min at 37 °C. Cells were then rinsed with 60% isopropanol for 15 s, followed by PBS washes.

### Protein secretion by ELISA

PCSK9 secretion from iPSCs and viSMCs was measured using a PCSK9 ELISA kit (Thermo Fisher Scientific) per the manufacturer's instructions. Similarly, secretion of ICAM-1 (Thermo Fisher Scientific, BMS201), VCAM-1 (Thermo Fisher Scientific, KHT0601), IL-6 (Thermo Fisher Scientific, 88-7066-77), and TNF-α (Thermo Fisher Scientific, 88-7346-77) from viECs were measured by corresponding ELISA kits per manufacturers' instructions.

### RNA extraction and qRT-PCR

Total RNA was extracted from iPSCs, viSMCs, and viECs using the RNeasy Mini Kit (Qiagen). The RNA concentration and purity were measured using a Nano-Drop Spectrophotometer. RNA was reverse transcribed into cDNA using the iScript cDNA Synthesis Kit (Bio-Rad) in an MWG-Biotech Primus 25 Thermal Cycler (Cole-Parmer). The resulting cDNA was then used in qRT-PCR reactions with iQ SYBR Green Supermix (Bio-Rad) and the primers listed in supplementary material Table 1 (Integrated DNA Technologies). Human U6 or GAPDH were used as reference genes and fold changes in expression were calculated using the ΔΔCt method.^69^ The PCR reaction was performed in a CFX Connect Real-Time PCR Detection System (Bio-Rad).

### TEBV fabrication

The linear and branched TEBVs were fabricated as previously described.^33^ High-concentration rat tail collagen (Corning) was diluted to 7 mg/ml using 10× Dulbecco's Modified Eagle's Medium (Sigma-Aldrich), 1 M sodium hydroxide (Sigma-Aldrich), and viSMCs in suspension at a density of 2 × 10^6^ cells/ml. The collagen mixture was injected into a set of molds surrounding a mandrel to create cylindrical gels with open lumens. The collagen gel was set at 37 °C for 1 h and then dehydrated using Kim Wipes (Whatman) to increase mechanical strength. The chamber was filled with viSMC media. The mandrels were removed, and the open lumen was seeded with viECs (9 × 10^6^ cells/ml). The whole device was rotated at 10 rotations/h for 1 h at 37 °C and then left in the incubator overnight. The following morning, the chamber was connected to two flow loops, one through the side ports and one through the TEBVs, connected to a media reservoir. The flow loops were connected to a peristaltic pump (Masterflex/Cole-Parmer) and linear TEBVs were perfused at a flow rate of 0.5 ml/min. For branched TEBVs, TEBVs with 80 ° branching angles between main and side outlets were used and operated as described previously.^34^ The pulsatile flow was obtained using a 1 Hz pulse-generator. The resulting oscillating flow characteristics were also reported previously.

### Preparation of eLDL, oxLDL, and TNF-α

Human plasma LDL (Lee Biosciences) was modified using a previously established method.^38^ The LDL protein concentration was measured using the Lowry Protein Assay (Thermo Fisher Scientific). For eLDL modification, based on the protein concentration, the LDL solution was incubated at 37 °C with different amounts of 0.05% trypsin (Sigma-Aldrich) and cholesterol esterase [Sigma-Aldrich, reconstituted to 1 mg/ml using 0.4 M potassium phosphate monobasic (Sigma-Aldrich)]. Briefly, LDL was first incubated with 7 *μ*g trypsin/mg LDL protein for 6 h, then 12 *μ*g cholesterol esterase/mg LDL protein for 10 h, then trypsin again at 24 *μ*g/mg LDL protein for 6 h, and finally 29 *μ*g cholesterol esterase/mg LDL protein for 48 h. Following the incubations, the LDL solution was dialyzed against PBS for 20–24 h.

For oxLDL modification, 5 mg of LDL was incubated in a 1 ml 10 *μ*M CuSO_4_ solution at 37 °C for 48 h. Following incubation, the modified LDL solution was dialyzed against PBS for 20–24 h.

TNF-α (Sigma-Aldrich or PeproTech) was reconstituted at 10 *μ*g/ml in sterile PBS, aliquoted, and frozen at −20 °C. Aliquots were used within 6 months of reconstitution.

### 3D immunofluorescence staining

TEBVs were disconnected from the peristaltic pump and gently perfused with PBS to remove any unadhered monocytes. For fixation, 4% paraformaldehyde was injected into the lumen of TEBVs and incubated for 10 min. Then, they were removed from the chamber and submerged in 4% paraformaldehyde for an additional 50 min. Following fixation, TEBVs were washed in PBS, permeabilized for 10 min using 0.1% TritonX-100 (Sigma-Aldrich), and blocked for 4–8 h at room temperature using 5% bovine serum albumin (Sigma-Aldrich) in 10% goat serum (Thermo Fisher Scientific). After blocking, TEBVs were incubated in primary antibodies overnight at 4 °C. The following antibodies were used for TEBV characterization: PCSK9 (Abcam—ab181142, 1:100), αSMA (Abcam—ab5694, 1:100), MYH11 (Santa Cruz Biotechnology—sc-6956, 1:100), CD31 (Abcam—ab28364, 1:100), VWF (Abcam—ab778, 1:100), ICAM-1 (Santa Cruz Biotechnology—sc-8439, 1:100), and VCAM-1 (Santa Cruz Biotechnology—sc-13160, 1:100). TEBVs were then incubated with secondary antibodies and Hoechst dye (1:500) for 2.5 h at room temperature. The following secondary antibodies were used: 488-goat anti-mouse IgG (Life Technologies, 1:200), 488-goat anti-rabbit IgG (Life Technologies, 1:200), and 594-goat anti-rabbit IgG (Life Technologies, 1:200). TEBVs were cut *en face* and placed between two coverslips. Images were acquired using either a Nikon model TE2000-U fluorescence microscope or Leica SP5 inverted confocal microscope.

### Monocyte perfusion and accumulation

Linear and branched TEBVs were perfused for seven days to mature. Then, they were treated with 10 *μ*g/ml eLDL and 50 ng/ml TNF-α for four days. On the final two days, monocytes (U937 or THP-1 for linear or branched TEBVs, respectively) were labeled with CellTracker^TM^ red-CMTPX or green CMFDA (Invitrogen) and perfused through the TEBVs at 1 × 10^6^ monocytes/ml. After two days, the lumens of the TEBVs were washed gently with PBS to remove any unadhered monocytes. TEBVs were then fixed with 4% paraformaldehyde as described in the 3D immunofluorescence staining section. Samples were cut *en face* and stained with Hoechst dye (1:500) for 2.5 h. Monocyte accumulation was determined by imaging TEBVs and counting the number of cells per area that colocalized cell tracker red and Hoechst dye.

In branched TEBVs, the resulting monocyte adhesion was significantly different at locations, i.e., inlets, main outlets, and side outlets, within a vessel as a result of focal adhesion phenomena characterized in branched arteries. Therefore, monocyte accumulation in branched TEBVs was depicted to indicate the corresponding locations.

### Chemical synthesis and analysis of small molecule PCSK9 inhibitor

The small molecule PCSK9 inhibitor, NYX-1492 (IUPAC name (5-{[(*R*)-3-Amino-4,4-difluoro-1-piperidyl]methyl} 3-[6-(trifluoromethyl)-3-pyridyl]phenylamino)(5-methyl-4-phenyl-2-pyridyl)formaldehyde) was discovered and developed by Nyrada, Inc. and synthesized at Shanghai ChemPartner Co. Ltd. (Chengdu, China). The compound was synthesized as the free base and was 100% pure as characterized by LCMS [supplementary material Fig. S5(b)]. All NMR spectra were recorded on a Bruker 400 (400 MHz) spectrometer [supplementary material Fig. S5(a)]. ^1^H chemical shifts are reported in δ values in ppm with the deuterated solvent as the internal standard. LCMS spectra were obtained on an Agilent 1200 series 6110 or 6120 mass spectrometer with electrospray ionization using the following conditions: Waters X Bridge C18 column (50 mm × 4.6 mm × 3.5 *μ*m), flow rate: 2.0 ml/min, and the column temperature: 40 °C.

### Preparation of small molecule PCSK9 inhibitor

NYX-1492 was reconstituted at a concentration of 10 mM in sterile DMSO (Corning), aliquoted, and stored at −20 °C. The final working concentration of the NYX-1492 compound for the treatment of cells and TEBVs was 8 *μ*M. We chose 8 *μ*M concentration of NYX-1492 based on results obtained by Nyrada (manuscript in preparation).

### Pharmacokinetic studies

Female C57BL/6 mice aged 10–14 weeks were purchased from Jihui Laboratory Animal Co. LTD and given free access to food and water throughout the in-life phase of the study. The animals were treated intravenously (5 mg/kg) or orally (50 mg/kg) with NYX-1492 formulated in 5% DMSO, 5% Solutol HS15, and 90% saline. Blood samples were taken at 0.083, 0.25, 0.5, 1, 2, 4, 8, and 24 h post-dose by restraining the animals manually and withdrawing approximately 110 *μ*l blood per time point via the facile vein. The samples were collected into pre-cooled EDTA-K2 tubes, placed on ice and subjected to centrifugation at 2000 × *g* for 5 min at 4 °C to obtain plasma. Plasma samples were stored at −70 °C until the day of analysis. The samples were assayed for their levels of NYX-1492 by LCMS using an API Triple Quad 6500+ System (SCIEX) operating in a positive electrospray ionization mode.

### Statistical analysis

Data were analyzed using one- or two-way ANOVA with a post-hoc Tukey test for multiple comparisons. A value of p < 0.05 was considered statistically significant. Data are represented as mean ± SEM, unless otherwise indicated.

## SUPPLEMENTARY MATERIAL

See the supplementary material for a complete primer list used for qPCR, guide RNA evaluation for both the PCSK9 activator and repressor, comparison of foam cell formation in human aortic SMCs using oxLDL and eLDL, the effect of PCSK9 activation or repression on TNF-α and IL-6 secretion from viSMCs, the pharmacokinetic profile of NYX-1492, the structure of NYX-1492, the effect of NYX-1492 on TNF-α and IL-6 secretion in viECs, the effect of NYX-1492 on monocyte adhesion in P+ branched TEBVs, the effect of PCSK9 activation alone on inflammatory cytokine expression in viECs and viSMCs, and elastic modulus of PAA gels used for the traction force microscopy analysis.

## Data Availability

The data that support the findings of this study are available from the corresponding author upon reasonable request.
